# Accelerometer-assessed sedentary work, leisure time and cardio-metabolic biomarkers during one year: Effectiveness of a cluster randomized controlled trial in parents with a sedentary occupation and young children

**DOI:** 10.1371/journal.pone.0183299

**Published:** 2017-08-24

**Authors:** Arto J. Pesola, Arto Laukkanen, Risto Heikkinen, Sarianna Sipilä, Arja Sääkslahti, Taija Finni

**Affiliations:** 1 Neuromuscular Research Center, Faculty of Sport and Health Sciences, University of Jyväskylä, Jyväskylä, Finland; 2 Faculty of Sport and Health Sciences, University of Jyväskylä, Jyväskylä, Finland; 3 Gerontology Research Center, Faculty of Sport and Health Sciences, University of Jyväskylä, Jyväskylä, Finland; Baker IDI Heart and Diabetes Institute, AUSTRALIA

## Abstract

**Background:**

It is unknown whether reducing sedentary time at work and during leisure time is possible and effective during one year.

**Methods:**

Office workers with young children were recruited for this one-year cluster-randomized controlled trial through kindergartens and primary schools from 7 clusters in the city of Jyväskylä, Finland. After a lecture, face-to-face tailored counseling was used to set contractually binding goals regarding reducing and breaking up sitting periods and increasing light intensity physical activity during work and leisure time. Primary outcomes of total, work and leisure sedentary time (<100 counts/min, cpm), light activity time (<2020 cpm), moderate-to-vigorous activity time (MVPA) and breaks/sedentary hour were assessed with a waist-worn Alive -accelerometer for 7 days, 5 times during the year. Anthropometrics (DXA), fasting biomarkers and self-reported diet were assessed as secondary outcomes. Data were collected between 2011–2013 and analyzed between 2013–2016 with a linear mixed-effects model fit by REML using likelihood ratio test and intention-to-treat–principle.

**Results:**

Participants from intervention (N = 71) and control (N = 62) regions were assessed at baseline and 117 completed the study. Sedentary leisure time decreased [-21.2 (95% CI -37.3 to -5.1) min/8 hours, likelihood ratio P<0.001] and light activity time [13.4 (-2.2 to 29.0) min/8 hours, P = 0.008] and breaks per sedentary hour [1.0 (-0.2 to 2.2), P = 0.010] increased in the intervention group as compared to controls at 3 months. The decrease in sedentary leisure time was maintained throughout the year [-7.9 (-24.0 to 8.3) min/8 hours, P = 0.029]. Small decreases in the control group’s work and leisure MVPA were observed mostly at 3 months. Small favorable intervention effects were observed for fasting plasma glucose at 3 months and for leg lean mass and apoB/apoA-1 ratio at 12 months, with no changes in other outcomes.

**Conclusions:**

Behavioral counseling induced a small decline in sedentary leisure time throughout one year in parents with a sedentary occupation and young children. Small concurrent changes in different biomarkers suggest that reducing sedentary leisure time during one year may be beneficial.

**Trial registration:**

ISRCTN28668090, registered 30 November 2011

## Background

A sedentary lifestyle is characterized by long periods of sitting throughout the day with relatively idle muscles resulting in low energy expenditure, insulin resistance and increased risk of chronic diseases [1]. An increasing proportion of the population is at an elevated risk of sedentary behavior owing to the increased prevalence of office work and sedentary leisure time habits, resulting in 9–11 h of total sitting time per day [2–4]. In people who sit more than 8 hours per day, more than 1.5 hours of moderate-intensity physical activity is required to offset the premature mortality risk attributable to their sitting time [5]. Such a high amount of moderate-intensity physical activity may be difficult to achieve, especially in people who face challenges to participate in physical activities. For example, working parents exhibit high levels of inactivity and cite family responsibilities, lack of time and work as barriers to physical activity [6,7]. Reductions in sedentary time may require less time commitment and be more easily incorporated in daily routines at work and home. Targeting sedentary behaviors may therefore be less prone to the barriers of physical activity and if proven effective, provide a feasible alternative to reap the health benefits.

Reducing sedentary time may be beneficial for health independent of engagement in moderate intensity physical activities. Acute laboratory-based studies have been effective at improving post-meal glucose tolerance and insulin sensitivity through frequent light activity bursts which have replaced sitting time [8]. Reducing prolonged sitting over the course of a day was found to be more effective for acute cardio-metabolic benefits than expending the same volume of energy in a single exercise bout, supporting the importance of performing light-intensity activities throughout the day [9]. The suggested mechanisms include improved muscle activity-induced insulin sensitivity and energy balance [8–10], which have the possibility to reduce ectopic fat accumulation and improve cardio-metabolic health in the long run [11]. However, the potential long-term benefits of reducing sedentary time are based on observational findings, and the evidence from long-term interventions is lacking.

Increasing evidence from mostly short-term intervention studies suggests that the strategies implemented to reduce sedentary time result in highly context-specific results for work or total sedentary time, but the sustainability of acute changes, especially outside of the workplace, are yet to be elucidated [11–14]. So far it is largely unclear whether intervention methods targeted separately at work and leisure sedentary time within the same study are effective in the short and long term. This is especially important given that the health risks of sitting during leisure time are more pronounced than those associated with work time sitting [15,16]. The purpose of this study was to test the effectiveness of behavioral counseling that included individual-level support aimed at reducing and breaking up sedentary time, and increasing light intensity physical activity during work and leisure time in parents with a sedentary occupation and young children. Work and leisure sedentary and physical activity time were assessed with waist-worn accelerometers five times during one year. Anthropometrics and cardio-metabolic biomarkers were measured and adjusted for moderate-to-vigorous activity and energy intake to elucidate potential health effects of reduced sedentary time. Cluster-randomization was used to avoid contamination of treatment within a small city. The short-term efficacy study of this intervention showed that this specific intervention reduced electromyography-derived muscle inactivity by 33 min a day without increasing moderate-to-vigorous muscle activity [17]. We hypothesized that this short-term effect would be maintained for a year and would be accompanied by improved lipid and glucose profiles.

## Methods

The methods for this cluster-randomized controlled trial have been reported earlier (InPACT, Actions to reduce sedentary time in parents and their young children [18]). The aim of this intervention was to counsel parents to decrease and break up their sedentary behavior, to increase non-exercise physical activity, and to increase their children’s physical activity. The effectiveness of the component targeting children’s behavior has been reported [19] and the present study reports the counseling process and the main outcomes in parents. The authors confirm that all ongoing and related trials for this intervention are registered. A delay in the registration of the trial was due to time constraints in the study implementation. Ethical approval for the project was received from the Ethics Committee of the Central Finland Health Care District on March 25, 2011 (Dnro 6U/2011) and the participants signed a written informed consent before participation. Reporting of this trial is guided by a checklist of the CONSORT 2010 Statement for reporting parallel group randomized trials [20].

### Setting, randomization and recruitment

**Fig 1:**
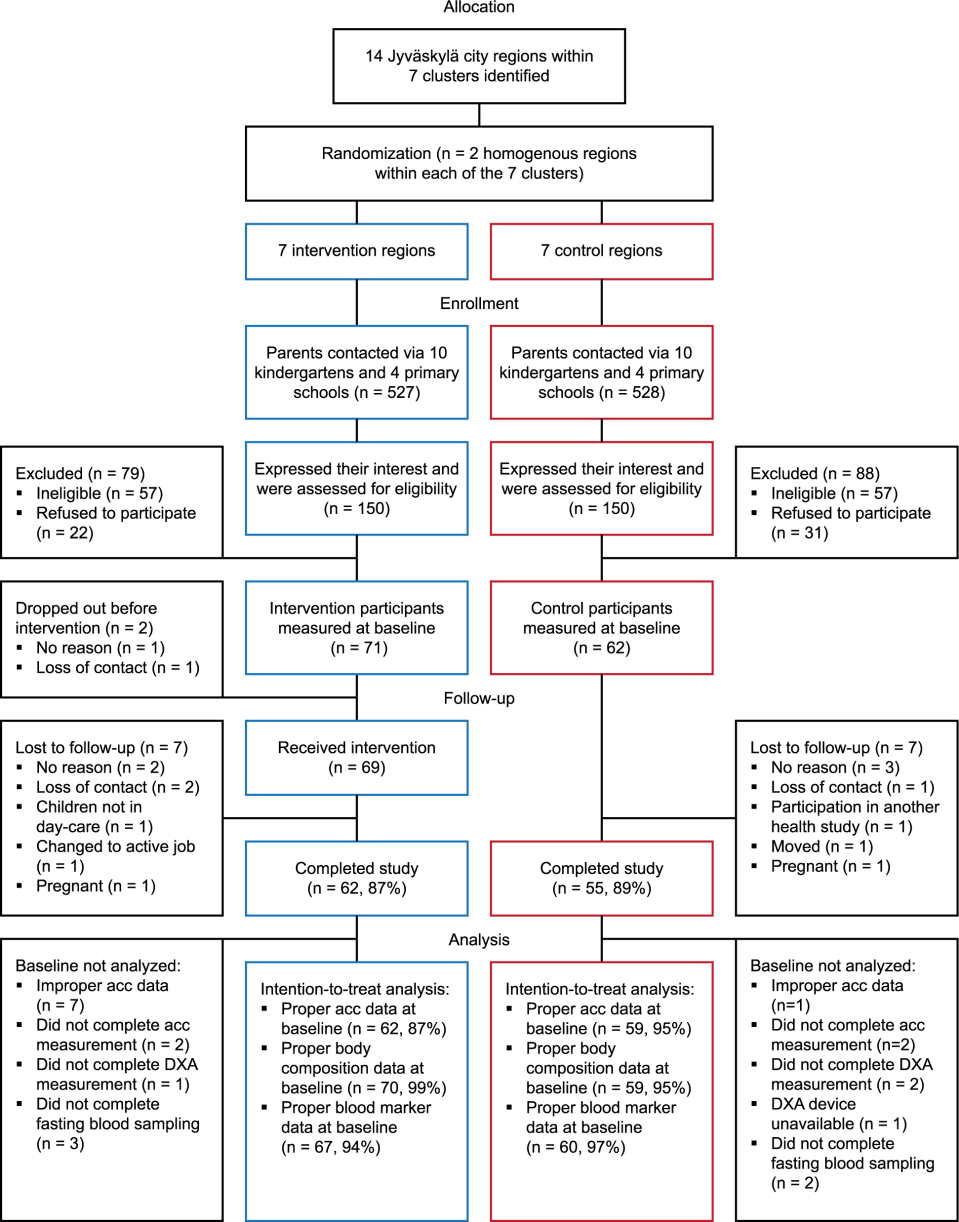
Flow chart of the study. Footnote: acc; accelerometer.

The flow chart of this cluster-randomized controlled trial is presented in Fig 1, and CONSORT as well as TIDieR checklists are provided as additional files (S1 Checklist and S2 Checklist). Sampling was performed in the city of Jyväskylä located in central Finland with a population of 133 000. Socioeconomic status and environmental possibilities for outdoor physical activities were identified in different city regions, resulting in 14 regions located within 7 homogenous clusters (2 homogenous counterpart regions within each cluster). The 14 regions were randomized by flipping a coin to select intervention (n = 7) and control (n = 7) regions within each cluster. Families from the intervention regions were recruited for the intervention group, and families from the control regions for the control group between the 1st of April, 2011 and the 30th of April, 2012. The 14 regions included a total of 8 primary schools and 21 kindergartens (2–5 schools or kindergartens per cluster). The recruitment began by delivering a total of 1055 recruitment forms to parents via the primary schools and kindergartens in spring 2011, autumn 2011 and spring 2012. Professional and health status, percent sitting time at work and contact information were obtained using these forms. In addition, general information about the study, inclusion and exclusion criteria, and an incentive to get diverse information about personal health, diet and physical activity and motor skills of their children were communicated. Inclusion criteria were: healthy men and women with at least one 3–8 year old child (one or both eligible parents were allowed to participate), parental occupation where they self-reportedly sit more than 50% of their work time, and children in all-day day-care in kindergarten or in the first grade of primary school. Exclusion criteria were: body mass index > 35 kg/m^2^ (calculated based on self-reported height and weight), self-reported chronic, long-term diseases, families with a pregnant mother at baseline, children with disorders that delay motor development, and concurrent participation in another study. No monetary incentive was offered to the participants. The researchers (AL, AP, TF) enrolled participants and performed randomization.

People fulfilling the inclusion criteria were contacted by phone and invited to one of 14 information lectures held in April 2011-April 2012, where the procedures were explained in detail and the first measurement time was scheduled. If people were unable to attend the lecture, details of the project were explained on the phone. Finally, a total of 133 participants were assessed at baseline between the 2nd of May, 2011 and the 2nd of May, 2012. After the baseline, measurements were conducted every 3 months until the one-year follow-up. Fig 1 summarizes the recruitment, randomization and analysis processes. Participants and staff were unblinded to the group allocation.

### Description of the intervention

The intervention program consisted of a lecture, face-to-face discussion including goal setting, and phone counselling (for one parent at a time). Childcare was offered during the counselling session, but children did not attend any intervention events. The behaviour change techniques used in the intervention were based on previous knowledge of effective interventions [21] and theory of planned behaviour [22]. Details of the intervention procedures have been reported previously [18].

The intervention lecture was given for a maximum of six participants at a time, within 2 weeks of the baseline measurements. During the 30 min lecture the health hazards of prolonged sitting and the challenges of the sitting-friendly modern environment from the adults’ perspective were described. It was underlined that the aim was to reduce and break up sitting time by increasing light intensity activities like light ambulation, as this is the easiest way to overcome the health hazards of prolonged sedentary time. The lecture was designed to provide information about the behaviour-health link, to provide information on consequences, to provide information about others’ approval, to provide instructions and general encouragement, and to emphasize identification as a role model for children [23].

The lecture was followed by face-to-face discussions with 1) one participant at a time when discussing work time behavior and 2) parents together when discussing leisure time behaviors, if both parents were participants. The participants were encouraged to think of feasible ways to decrease and break up sitting time during these routines and to increase light-intensity physical activities, which were then formulated to small step progressive goals for each participant. The specific goals for each domain were written down into an agreement document that was signed by the participant and the researcher. The researcher transcribed the goals to a certificate, which was subsequently delivered to the participant. The underlying theoretical frameworks were motivational interviewing in order to provide general encouragement and instructions, to prompt intention formation and specific goal setting, and to agree on a behavioural contract [23]. The lecture and individual discussions were led by researchers (AL, AP, TF) who had all undergone an orientation about good practices in PA counseling.

To promote compliance with the goals, a phone discussion with each participant was performed after two and five months of counselling. Participants were asked to self-evaluate the implementation of the goals (prompt review of behavioural goals). After this, perceived barriers were discussed and possible modifications to the goals were made (prompt barrier identification, provide instructions, prompt specific goal setting). The behavior change techniques described here were specific to parents’ sedentary and light activity time and the techniques regarding children’s behavior are reported elsewhere [19].

Participants in the intervention group were encouraged to maintain the intended behavior after the end of the counseling period. The last 6 months of the study were identical for both groups, consisting of 9 and 12 month assessments without any counseling. At 12 months, after the follow-up assessments were completed, the participants in the control group received a shortened version of the counseling.

### Protocol and data

The protocol included data collection at the research laboratory of the University of Jyväskylä, and measurements of sedentary time and physical activity during normal daily life. Each measurement was started at the research laboratory, where the participants arrived in the morning in fasted state before going to work. At baseline, six and twelve months, body composition and blood pressure were measured first followed by blood sampling, whereas at three and nine months only blood sampling was performed. The protocol continued with the attachment of the accelerometer and breakfast, where instructions for wearing the accelerometer for seven days and for filling in questionnaires were given. At baseline a short activity test battery was performed for purposes of EMG measurement and has been reported elsewhere [17]. After the laboratory measurements the participants left for work and were expected to wear the accelerometer and to fill in an activity diary. After the measurement period the accelerometer and the activity diary were to be returned to the research laboratory or to a box located at the kindergarten where the initial recruitment was performed.

#### Primary outcomes

Primary outcomes of sedentary, light and moderate-to-vigorous activity time and breaks/sedentary hour were assessed with a waist-worn two-dimensional accelerometer (dynamic range ± 2.7g, sample rate 75 / s, resolution 8 bit, bandwidth 0–20 Hz, manufacturer Alive Technologies Ltd., Australia) at baseline and 3, 6, 9 and 12 months thereafter. This light-weight device was worn in a firm elastic band on the anterior right side of the waistline for 7 days at each measurement point. During this period the participants kept a log sheet of their working times, bed times and any abnormal behaviors which could possibly affect the measurements. These included e.g. bathing, water-based activities or contact-sports, during which the device was detached. The device was also removed at night. Based on the diary, data were analyzed for total wear time, work time and weekday and weekend day leisure time excluding non-wear time. A wear time of at least 10 hours was required for valid week and weekend days [24,25]. Requirements for a valid total week were a minimum of two days measurement including at least one weekday resulting in a minimum reliability of 0.68 (S1 Supporting information). Work and leisure time were identified based on diaries, and two valid measurement days were required for both. To improve comparability and to minimize the variation in wear time, the outcomes were normalized to 16 hours waking time (total and weekends) or 8 hours (work and leisure time) [13]. To enable use of validated count thresholds, which are specific to a given monitor model, we performed calibration measurements. A device-specific factor used in the analysis was derived from simultaneous recordings with the Alive monitor and ActiGraph GT3X monitor (Actigraph LCC, Pensacola, FL, USA) in a custom-made calibration machine (University of Jyväskylä) and in two adults during normal daily life conditions. Time spent in sedentary (< 100 counts/min [24]), light (≥ 100 < 2020 counts/min) and moderate-to-vigorous activity (≥ 2020 counts/min [25]) were analyzed in one minute epochs. A break in sedentary time was defined as an interruption in sedentary time when accelerometer counts rose up to or above 100 counts/min for a minimum of one-minute. The number of breaks was then normalized to sedentary time (h) yielding breaks/sedentary hour.

#### Secondary outcomes

The secondary outcomes included cardio-metabolic health markers, energy intake and diet composition. The measurement of cardio-metabolic health markers was done at the research laboratory of the University of Jyväskylä. Before arriving to the laboratory in the morning participants were asked to fast for a minimum of 10 hours and refrain from vigorous exercise the day before. Subjects’ height and weight were measured with standard procedures. Blood pressure was measured twice from the left arm in supine position after a five minute rest (Omron M6W, Omron Healthcare Co., Ltd. Kyoto, Japan). Body composition was measured with dual-energy X-ray absorptiometry (DXA, LUNAR Prodigy, GE Healthcare). Professional laboratory personnel measured and analyzed total lipids, glucose and insulin with standardized procedures (Konelab 20 XTi analyzer, Thermo-Fisher, Espoo, Finland). Homeostasis model assessment (HOMA) was used to calculate hepatic insulin resistance (HOMA-IR) and basal insulin secretion (HOMA-%B) from fasting glucose and insulin [26]. Additionally, all serum samples were analyzed using a high- throughput serum NMR metabolomics platform [27] for apolipoproteins and mean diameter of lipoproteins.

Energy intake and diet composition were assessed from dietary records in which the participants were asked to report all foods and the quantities consumed. The records were kept on three weekdays and on one weekend day at the baseline and end of the study, and on one weekday at 3, 6 and 9 months of the study. Nutri Flow software (Nutri Flow Oy, Oulu, Finland) was used to analyze intakes of total energy and energy-yielding nutrients as a percentage of total energy intake.

### Sample size

The a priori planned sample size has been reported previously [28]. However, we recalculated the sample size based on the initial efficacy study of this intervention [17]. A minimum difference of interest (MDI) was 30 minutes of total sedentary time, which was expected to induce significant cardio-metabolic and anthropometric benefits [11,17]. Based on the efficacy study, a sample size of 36 in each group was required to achieve ≥80% power at 5% two-tailed significance to detect decreased total muscle inactivity time. The sample sizes required to achieve a similar power for work and leisure time were 25 and 55 for each group, respectively. These sample sizes provided similar power for muscle light activity time and number of bursts. Assuming a small cluster effect (1.05) and 10% attrition, 64 participants per group was the target sample size.

### Statistical analyses

Intervention effectiveness was tested with linear mixed-effects model fit by REML in statistical programming language R (R 3.0.1, NLME package, the R foundation for Statistical Computing). The analysis was based on a three level hierarchy, where the random grouping variables participants (n = 133) were nested within families (n = 89), and families were nested within the clusters (n = 7). Likelihood ratios were used to test the effectiveness from baseline to every measurement time point separately (3 months: from baseline to 3 months; 6 months: from baseline to 3 and 6 months; etc. up to 12 months). Intention-to-treat principle was used meaning that all participants with acceptable baseline data, including those who dropped out later or had missing data, were retained in the analysis. Missing data and attrition were assumed to occur at random and background characteristics between completers and those lost to follow-up were compared with Chi-square and Mann-Whitney U test and are reported. Estimated marginal means are presented for an unadjusted model. P-values are presented for the unadjusted model and a model adjusted for baseline values of the dependent variable, measurement duration (in case of accelerometer-derived variables), starting season (spring/summer/autumn/winter), worktime/day, number of children, single parent (yes/no), age and sex (adjusted P-value). The models testing the effects on health outcomes were additionally adjusted for MVPA and energy intake. Log-transformation was used where required but non-transformed estimated marginal means are presented. As part of the intervention evaluation, three-way interaction terms were used to study the influence of gender (Group x Time x Gender), single/two participating parents (Group x Time x No of participating parents) and season at baseline (Group x Time x Season) on intervention effectiveness.

## Results

### Participants

A total of 300 individuals (150 from intervention and 150 from control regions) expressed an interest in the study by returning the recruitment form and were assessed for eligibility (Fig 1). From these, 71 participants from the intervention (47%) and 62 from the control (41%) regions (9.5 ± 3.4 participants per region) met the inclusion criteria and were assessed at baseline. Of the InPACT study participants, 71% had university-level education, compared to a mean of 35% across the recruitment regions. The participants were young adults (age range 28–53 years) of whom almost half were working as managerial employees (Table 1). Based on objective measurements, about 62% of the whole day, 80% of work time, 47% of leisure time and 58% of weekends were spent in sedentary behaviors (Table 1). The secondary outcomes at baseline are presented in S1 Table.

**Table 1:** Baseline characteristics of intervention and control group participants.

	Intervention		Control	
	n		n	
Two parents vs one parent from a family participated, %	71	31.0	62	22.6
Women, %	71	60.0	62	51.6
Age, years	71	36.6 (5.1)	62	39.6 (5.3)
Height, cm	71	170.9 (9.8)	60	171.3 (8.2)
Body mass, kg	71	72.0 (15.4)	60	71.8 (14.0)
No. of children	53	1.3 (0.5)	42	1.6 (0.7)
Worktime, hours/week	71	36.1 (6.6)	61	38.1 (4.0)
Professional status	69		61	
Employee, n (%)		14 (20)		16 (26)
Official, n (%)		11 (16)		11 (18)
Managerial employee, n (%)		33 (47)		28 (45)
Entrepreneur, n (%)		4 (6)		3 (5)
Other, n (%)		7 (10)		3 (5)
No data, n (%)		1 (1)		1 (2)
Accelerometer-derived variables	n		n	
Total days	62	5.5 (1.4)	59	5.8 (1.4)
Total duration / day (min)	62	862.0 (48.0)	59	875.2 (76.1)
Total sedentary time (min / 16h)	62	545.1 (81.1)	59	525.7 (79.7)
Total light activity time (min / 16h)	62	382.2 (76)	59	400.4 (80.7)
Total moderate-to-vigorous activity time, (min / 16h)	62	34.1 (19.9)	59	35.4 (18.3)
Total breaks per sedentary hour	62	10.2 (2.5)	59	10.3 (2.6)
Work days	54	3.9 (1.2)	58	4.1 (1.2)
Work duration / day (min)	54	409.3 (62.9)	58	429.8 (76.9)
Work sedentary time (min / 8h)	54	338.2 (62.9)	58	334.8 (63.8)
Work light activity time (min / 8h)	54	130.4 (62.1)	58	133.1 (61.5)
Work moderate-to-vigorous activity time, (min / 8h)	54	12.0 (10.0)	58	12.8 (8.8)
Work breaks per sedentary hour	54	8.6 (5.0)	58	8.2 (4.3)
Leisure days	59	4.2 (1.1)	58	4.5 (1.1)
Leisure duration / day (min)	59	495.5 (113.3)	58	475.8 (94.1)
Leisure sedentary time (min / 8h)	59	238.9 (38.7)	58	221.5 (39.7)
Leisure light activity time (min / 8h)	59	221.9 (39.6)	58	235.5 (38.5)
Leisure moderate-to-vigorous activity time, (min / 8h)	59	20.4 (14.0)	58	24.2 (17.3)
Leisure breaks per sedentary hour	59	12.0 (2.6)	58	13.1 (3.1)
Weekend days	55	1.6 (0.5)	50	1.8 (0.4)
Weekend duration / day (min)	55	794.2 (95.0)	50	814.7 (100.0)
Weekend sedentary time (min / 16h)	55	478.4 (120.5)	50	458.7 (117.6)
Weekend light activity time (min / 16h)	55	449.0 (117.6)	50	470.3 (121.1)
Weekend moderate-to-vigorous activity time, (min / 16 h)	55	34.8 (31.4)	50	32.5 (27.4)
Weekend breaks per sedentary hour	55	11.9 (3.8)	50	12.2 (3.7)

Data presented as mean and standard deviation (SD) unless otherwise noted.

Between the baseline and 12-month follow-up, seven participants dropped out from the control group (11%) and nine from the intervention group (13%), two of whom (3%) withdrew before the intervention (Fig 1). Between those who adhered to the study and those who dropped out, there were no differences at baseline in the number of families where two vs one parent participated (28.2% / 18.8%), gender (women 56% / 62%), age (37.9 ± 5.4 y / 38.7 ± 5.6 y), worktime/week (37.2 ± 5.6 h / 35.8 ± 6.0 h), professional status distribution (data not shown), BMI (24.4±3.6 kg/m^2^ / 25.1±5.0 kg/m^2^) or accelerometer-assessed sedentary time (538.1 ± 80.5 min per 16h / 507.6 ± 81.8 min per 16h) and MVPA (34.7 ± 19.2 min per 16h / 35.4 ± 17.8 min per 16h). The number of participants with valid accelerometer data in each domain, number of days and measurement time per day are reported in Table 1. No significant group x time–interactions were observed. Valid accelerometer data were available from 547 (90%) whole day measurements throughout the year while 42 (7%) were missing due to dropouts and 16 (3%) due to improper measurement. Similarly, valid data were obtained from 85%, 90% and 69% of the worktime, weekday leisure time and weekend day measurements, respectively, with reasons for data exclusion including improperly filled diaries making domain separation impossible, problems with measurement, or drop-out.

### Effectiveness of the intervention

**Fig 2:**
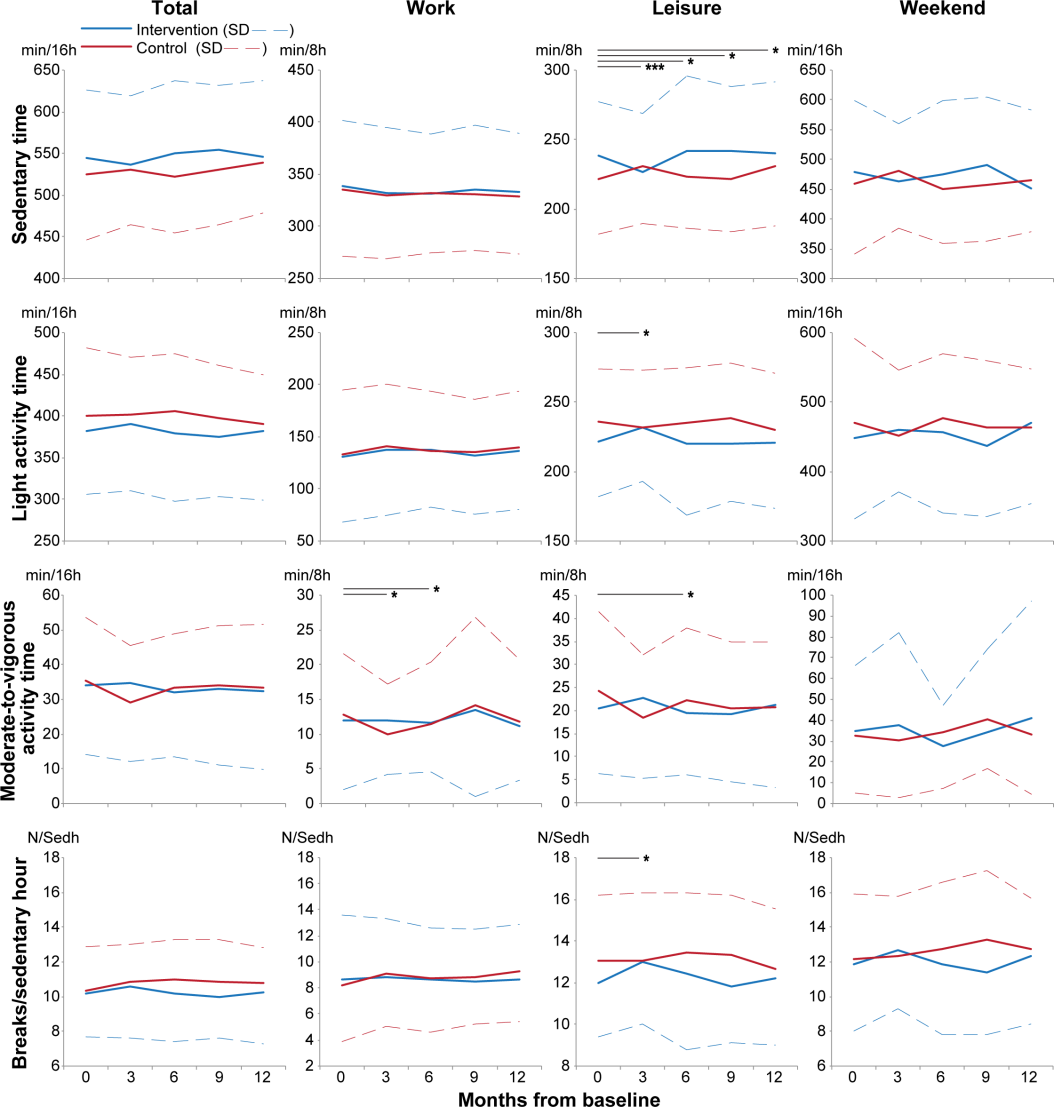
Intervention effectiveness on absolute values of primary outcomes in different domains throughout the study. Means and standard deviations (SD) are presented. Footnote: Significance for likelihood ratios between models with and without group x time interaction at different time periods is illustrated as follows: * = P < 0.05, *** = P < 0.001.

Effectiveness of the intervention on the primary outcomes is presented as absolute values in Fig 2 and as estimated marginal means of change from baseline in Table 2. During work time a group x time interaction was observed for moderate-to-vigorous activity at three (P = 0.011) and six months (P = 0.015). The change at three months was still evident after the full adjustments. Work moderate-to-vigorous activity changed between groups by 2.8 minutes / 8 hours at three months (P = 0.034) and by 1.1 minutes / 8 hours at six months (P = 0.014), both driven by decreases within the control group.

**Table 2:** Intervention effectiveness on primary outcomes in different domains as unadjusted estimated marginal means of change from baseline.

			Mean change (95% CI)			Mean difference in change (95% CI)		
	Time	n	Intervention	n	Control	Intervention—Control	P	Adj. P
Total								
Sedentary (min/16 h)	3m	57	-12.2 (-31.2 to 6.9)	54	2.8 (-16.8 to 22.4)	-15.0 (-42.3 to 12.3)	0.12	0.39
	6m	53	7.1 (-12.4 to 26.6)	53	-4.2 (-23.9 to 15.5)	11.3 (-16.4 to 39.1)	0.94	0.25
	9m	52	3.3 (-16.4 to 22.9)	51	4.3 (-15.6 to 24.3)	-1.0 (-29.0 to 26.9)	0.36	0.78
	12m	54	-1.0 (-20.4 to 18.4)	52	10.4 (-9.5 to 30.2)	-11.4 (-39.1 to 16.4)	0.37	0.79
Light (min/16 h)	3m	57	12.2 (-6.2 to 30.5)	54	3.4 (-15.4 to 22.3)	8.7 (-17.6 to 35.1)	0.14	0.36
	6m	53	-4.1 (-22.9 to 14.7)	53	6.7 (-12.2 to 25.7)	-10.8 (-37.5 to 15.9)	0.81	0.21
	9m	52	-2.7 (-21.6 to 16.2)	51	-2.4 (-21.6 to 16.8)	-0.3 (-27.3 to 26.6)	0.60	0.91
	12m	54	3.2 (-15.5 to 21.9)	52	-8.2 (-27.3 to 10.9)	11.3 (-15.4 to 38.1)	0.52	0.84
Moderate-to-vigorous (min / 16 h)	3 m	57	0.2 (-4.6 to 4.9)	54	-6.3 (-11.2 to -1.5)	6.5 (-0.3 to 13.3)	0.48	0.44
	6 m	53	-2.8 (-7.7 to 2)	53	-2.6 (-7.4 to 2.3)	-0.3 (-7.1 to 6.6)	0.08	0.12
	9 m	52	-0.5 (-5.3 to 4.4)	51	-1.8 (-6.8 to 3.1)	1.4 (-5.6 to 8.3)	0.13	0.40
	12 m	54	-1.7 (-6.6 to 3.1)	52	-2.1 (-7.1 to 2.8)	0.4 (-6.5 to 7.3)	0.19	0.39
Breaks/Sedentary hour	3m	57	0.6 (0 to 1.2)	54	0.6 (0.0 to 1.2)	0.0 (-0.9 to 0.9)	0.53	0.79
	6m	53	0.0 (-0.6 to 0.6)	53	0.7 (0.1 to 1.3)	-0.7 (-1.6 to 0.2)	0.52	0.86
	9m	52	0.0 (-0.6 to 0.7)	51	0.5 (-0.1 to 1.2)	-0.5 (-1.4 to 0.4)	0.32	0.81
	12m	54	0.2 (-0.4 to 0.9)	52	0.5 (-0.1 to 1.1)	-0.3 (-1.2 to 0.6)	0.43	0.88
Work time								
Sedentary (min/8 h)	3m	52	-8.5 (-24 to 7.0)	54	-5.5 (-20.6 to 9.7)	-3.1 (-24.8 to 18.6)	0.86	0.80
	6m	51	-8.4 (-24 to 7.2)	53	-3.9 (-19.2 to 11.4)	-4.5 (-26.3 to 17.3)	0.68	0.37
	9m	47	-5.8 (-21.7 to 10.1)	52	-5.6 (-21.0 to 9.7)	-0.2 (-22.3 to 21.9)	0.97	0.92
	12m	53	-6.7 (-22.1 to 8.7)	51	-6.5 (-22.0 to 8.9)	-0.1 (-22.0 to 21.7)	0.99	0.95
Light (min/8 h)	3m	52	8.8 (-5.9 to 23.5)	54	8.4 (-6 to 22.8)	0.4 (-20.2 to 21)	0.95	0.80
	6m	51	7.0 (-7.8 to 21.8)	53	5.6 (-8.9 to 20.1)	1.4 (-19.3 to 22.1)	0.71	0.36
	9m	47	4.4 (-10.7 to 19.5)	52	4.1 (-10.4 to 18.7)	0.3 (-20.7 to 21.2)	1.00	0.95
	12m	53	7.6 (-7 to 22.3)	51	7.5 (-7.1 to 22.2)	0.1 (-20.6 to 20.8)	1.00	0.98
Moderate-to-vigorous (min / 8 h)	3 m	52	-0.1 (-2.8 to 2.6)		-2.9 (-5.5 to -0.3)	2.8 (-0.9 to 6.6)*	**0.011**	**0.006**
	6 m	51	-0.5 (-3.2 to 2.2)		-1.6 (-4.2 to 1.0)*	1.1 (-2.7 to 4.9)*	**0.015**	0.06
	9 m	47	1.4 (-1.3 to 4.2)		1.4 (-1.2 to 4.0)	0.0 (-3.8 to 3.8)	0.09	0.22
	12 m	53	-0.8 (-3.5 to 1.9)		-1.0 (-3.7 to 1.6)	0.2 (-3.6 to 4.0)	0.09	0.28
Breaks/Sedentary hour	3m	52	0.4 (-0.7 to 1.4)	54	1.0 (0.0 to 2.0)*	-0.7 (-2.1 to 0.8)	0.48	0.56
	6m	51	0.1 (-1.0 to 1.1)	53	0.8 (-0.2 to 1.8)	-0.7 (-2.1 to 0.8)	0.93	0.50
	9m	47	0.1 (-1.0 to 1.1)	52	0.8 (-0.2 to 1.8)	-0.7 (-2.2 to 0.8)	0.70	0.73
	12m	53	0.1 (-0.9 to 1.1)	51	1.2 (0.2 to 2.2)*	-1.1 (-2.5 to 0.4)	0.68	0.70
Leisure time								
Sedentary (min/8 h)	3m	55	-11.3 (-22.7 to 0.0)	55	9.9 (-1.5 to 21.3)	-21.2 (-37.3 to -5.1)**	**<0.001**	**0.004**
	6m	55	2.9 (-8.4 to 14.3)	53	2.0 (-9.5 to 13.5)	0.9 (-15.3 to 17.1)	**0.022**	0.14
	9m	51	2.3 (-9.3 to 13.9)	52	1.2 (-10.5 to 12.8)	1.1 (-15.3 to 17.6)	**0.014**	0.20
	12m	56	0.8 (-10.5 to 12.1)	52	8.6 (-3.0 to 20.2)	-7.9 (-24.0 to 8.3)	**0.029**	0.26
Light (min/8 h)	3m	55	9.3 (-1.7 to 20.3)	55	-4.1 (-15.2 to 7.0)	13.4 (-2.2 to 29.0)	**0.008**	**0.019**
	6m	55	-1.8 (-12.8 to 9.2)	53	-0.1 (-11.3 to 11.0)	-1.7 (-17.4 to 14.0)	0.09	0.57
	9m	51	-1.7 (-13 to 9.6)	52	2.9 (-8.4 to 14.2)	-4.6 (-20.5 to 11.4)	0.12	0.44
	12m	56	-1.2 (-12.1 to 9.8)	52	-4.9 (-16.1 to 6.4)	3.7 (-12 to 19.4)	0.20	0.52
Moderate-to-vigorous (min / 8 h)	3 m	55	2.2 (-1.8 to 6.2)	55	-5.8 (-9.8 to -1.8)*	8.0 (2.3 to 13.6)*	0.08	0.38
	6 m	55	-0.9 (-4.9 to 3.1)	53	-1.9 (-5.9 to 2.2)	1.0 (-4.7 to 6.7)	**0.039**	0.20
	9 m	51	-0.5 (-4.6 to 3.6)	52	-4.0 (-8.0 to 0.1)	3.4 (-2.4 to 9.2)	0.09	0.29
	12 m	56	0.9 (-3.1 to 4.9)	52	-3.6 (-7.7 to 0.5)	4.5 (-1.2 to 10.2)	0.13	0.54
Breaks/Sedentary hour	3m	55	1.0 (0.1 to 1.9)	55	0.0 (-0.9 to 0.9)	1.0 (-0.2 to 2.2)	**0.010**	**0.017**
	6m	55	0.5 (-0.4 to 1.3)	53	0.4 (-0.5 to 1.3)	0.0 (-1.2 to 1.3)	0.34	0.40
	9m	51	-0.1 (-1.0 to 0.8)	52	0.3 (-0.6 to 1.1)	-0.4 (-1.6 to 0.9)	0.18	0.38
	12m	56	0.3 (-0.6 to 1.1)	52	-0.3 (-1.2 to 0.6)	0.6 (-0.6 to 1.8)	0.21	0.53
Weekends								
Sedentary (min/16 h)	3m	43	-8.7 (-43.8 to 26.4)	42	21.9 (-14.1 to 57.9)	-30.6 (-80.9 to 19.6)	0.11	0.11
	6m	35	3.7 (-33.9 to 41.3)	41	-8.1 (-44.3 to 28.2)	11.8 (-40.5 to 64.0)	0.48	0.53
	9m	39	11.9 (-24.3 to 48.2)	34	-0.6 (-39.1 to 37.9)	12.5 (-40.4 to 65.4)	0.32	0.20
	12m	40	-22.9 (-58.8 to 13.0)	38	2.7 (-34.5 to 39.8)	-25.6 (-77.2 to 26.1)	0.36	0.31
Light (min/16 h)	3m	43	4.1 (-30 to 38.1)	42	-18.7 (-53.6 to 16.2)	22.8 (-25.9 to 71.5)	0.07	0.10
	6m	35	0.0 (-36.4 to 36.5)	41	6.6 (-28.6 to 41.7)	-6.5 (-57.2 to 44.1)	0.73	0.66
	9m	39	-12.6 (-47.7 to 22.6)	34	-6.1 (-43.5 to 31.3)	-6.5 (-57.8 to 44.8)	0.60	0.37
	12m	40	16.7 (-18.1 to 51.5)	38	-3.0 (-39.0 to 33.0)	19.7 (-30.4 to 69.8)	0.68	0.59
Moderate-to-vigorous (min / 16 h)	3 m	43	4.8 (-6.9 to 16.5)	42	-3.5 (-15.5 to 8.6)	8.3 (-8.5 to 25.0)	0.29	0.42
	6 m	35	-3.9 (-16.4 to 8.5)	41	1.9 (-10.3 to 14)	-5.8 (-23.2 to 11.6)	0.82	0.97
	9 m	39	2.0 (-10.4 to 14.3)	34	6.8 (-6.0 to 19.5)	-4.8 (-22.6 to 12.9)	0.10	0.14
	12 m	40	7.4 (-4.7 to 19.6)	38	0.1 (-12.2 to 12.4)	7.4 (-9.9 to 24.7)	0.20	0.22
Breaks/Sedentary hour	3m	43	0.6 (-0.6 to 1.9)	42	0.0 (-1.2 to 1.3)	0.6 (-1.1 to 2.4)	0.09	0.09
	6m	35	-0.3 (-1.6 to 1.0)	41	0.6 (-0.6 to 1.9)	-0.9 (-2.7 to 0.9)	0.43	0.75
	9m	39	-0.5 (-1.7 to 0.8)	34	0.9 (-0.5 to 2.2)	-1.3 (-3.2 to 0.5)	0.18	0.10
	12m	40	0.3 (-0.9 to 1.5)	38	0.5 (-0.8 to 1.8)	-0.2 (-2.0 to 1.6)	0.28	0.16

P-values indicated as follows

* < 0.05 and

** < 0.01.

Group x time interaction P-values are based on likelihood ratios. P = unadjusted P-value, Adj. P = P-value adjusted for age, sex, baseline value, season at baseline (spring/summer/autumn/winter), work time/week, number of children and marital status (single/relationship). P-values for moderate-to-vigorous activity performed for log-transformed data but non-transformed estimated marginal means are presented.

During leisure time, group x time interactions were significant for sedentary time at three (P < 0.001), six (P = 0.022), nine (P = 0.014) and 12 months (P = 0.029), for light activity time (P = 0.008) and breaks/sedentary hour at three months (P = 0.010) and for moderate-to-vigorous activity at six months (P = 0.039). All changes at three months were still evident after the full adjustment. At three months, sedentary leisure time decreased 21.2 minutes / 8 hours more in the intervention group than the control group (P = 0.010). At the same time, leisure moderate-to-vigorous activity increased 8.0 minutes more in the intervention group than in controls (P = 0.037) due a decrease within the control group (P = 0.017). No changes at other times or in other domains were observed (Table 2 and Fig 2).

### Secondary outcomes

Intervention effectiveness on energy intake and diet composition (S2 Table), anthropometrics and blood pressure (S3 Table) and blood-drawn cardio-metabolic biomarkers (S4 Table) are presented in Supplementary Tables. No group x time effects on energy intake or diet composition were observed. Fasting plasma glucose changed significantly at three months (P = 0.021), and HOMA-%B changed significantly at three (P = 0.042) and six months (P = 0.046) in the intervention compared to the control group. Only HOMA-%B remained independent of full adjustment, including moderate-to-vigorous activity and energy intake. The change in fasting plasma glucose was different between groups at three (-0.18, 95% CI -0.35 to 0.00 mM, P = 0.049) and nine months (-0.22, 95% CI -0.4 to -0.03 mM, P = 0.020), driven by larger decreases within the intervention group than the control group. Insignificant group effects were observed for HOMA-%B at all time points.

At 12 months, the group x time effects were significant for the ratio of apoB to apoA-1 (P = 0.039) and for leg lean mass (P = 0.021). Only the change in ratio of apoB to apoA-1 remained independent of full adjustment. Leg lean mass changed between groups at 12 months (0.48, 95% CI 0.18 to 0.77%, P = 0.002), where a decrease within the control group was observed (P < 0.001, S3 Table). The ratio of apoB to apoA-1 changed between groups at 12 months (-0.04, 95% CI -0.07 to -0.01, P = 0.002), driven by a decrease within the intervention group (P = 0.033) and an increase within the control group (P = 0.027, S4 Table). After adjustment, the group x time changes in HDL cholesterol became significant at three, nine and 12 months, and in total cholesterol at 12 months, but there were no changes between the groups at any time points. No changes in other biomarkers were observed.

### Intervention evaluation

The lecture and tailored counseling session were attended by 69 parents (97% of those measured at baseline). Parents set an average of 3.3 ± 2.1 goals for work time (83% set a goal), 2.3 ± 1.6 goals for leisure time (96% set a goal) and 1.9 ± 1.8 goals for weekends (68% set a goal). The most commonly mentioned topic regarding work time goals was related to breaking up prolonged sitting periods, e.g. to stand up from a chair every half an hour (mentioned by 53% of responders). During leisure time the most commonly mentioned topic was related to increasing light intensity physical activity during commuting, e.g. by leaving the car further away and walking to work (mentioned by 48% of responders). During weekends, going outside to increase light intensity activity instead of sitting inside was the most common topic (mentioned by 81% of responders). At 2 and 5 months, 64 (90%) and 51 (72%) parents were reached for phone discussion, respectively.

Gender affected the intervention effectiveness in total breaks/sedentary hour (P = 0.013) and in work light activity time (P = 0.037). Total breaks/sedentary hour changed in the intervention group compared to the control group by 2.8 (95% CI -4.6 to -1.0, P = 0.002) and 2.1 (-3.9 to -0.3, P = 0.024) less in males than females at 3 and 12 months, respectively. Work light activity time changed in the intervention group compared to the control group 58.7 min/8h (-100.1 to -17.3, P = 0.006) less in males than females at 3 months.

Intervention effectiveness differed between single vs. two participating parents in total (P = 0.037) and leisure (P = 0.004) moderate-to-vigorous activity time. Total moderate-to-vigorous activity time changed in the intervention group compared to the control group 5.4 min/16 h (-8.4 to 19.1, P = 0.034) more in two than single participating parents at 3 months. However, during leisure time moderate-to-vigorous activity time changed in the intervention group compared to the control group 16.3 min/16h (-27.9 to -4.7, P = 0.030) less in two compared to single participating parents at 9 months. No effect of season on intervention effectiveness was observed.

## Discussion

Despite the fact that several observational studies have identified sedentary behavior as a prevalent and independent cardio-metabolic risk factor, evidence regarding the potential to modify primarily sedentary behavior in different domains of daily life is limited to short-term interventions [14]. This one year cluster randomized controlled trial was designed to decrease sedentary time and increase light activity time at work and during leisure time in parents with a sedentary occupation and young children. The lecture, tailored counseling and two follow-up calls during the first six months induced a small beneficial intervention effect on weekday sedentary leisure time throughout the whole year. At the same time, some small positive changes in biomarkers were observed, supporting a potential causal effect of reduced sedentary leisure time on improved metabolic profile. This cluster randomized controlled trial is one of the first to target sedentary behaviors in ecologically valid settings across several domains, and suggests that both behavioral and physiological benefits of reduced sedentary time are domain-specific.

The present results emphasize the incidental and highly domain-specific nature of habitual sedentary time. In contrast to the hypothesis, the initial decrease in total sedentary time [17] was not maintained throughout the year, and the effects were seen only in leisure time. Moreover, investigation of the timeline trend suggests that the intervention participants were able to reduce leisure sedentary time in the first months of the study, followed by a modest increase towards midline, and then maintained this level until the end of the study. The trend in the control group participants was almost the opposite at the beginning, accompanied by a modest increasing trend in sedentary leisure time towards the end of the study, resulting in a beneficial intervention effect. Even though a recent meta-analysis reported that studies targeting specifically sedentary time induced a 48 minute decrease in daily sedentary time, none of them studied whether this short-term effect could be maintained [14]. The results of this 12 month study suggest that although the initial efficacy study showed a positive acute effect [17], intervention methods that are successful in the short term do not necessarily induce long-term positive effects in total sedentary time, while long-term benefits may be domain-specific.

Sitting is a predominant activity in multiple domains of daily life and interventions targeting reduced sedentary time in these domains should be highly context-specific. Although the present intervention was designed to change both work time and leisure time behaviors, the only changes were seen in sedentary leisure time, for many possible reasons. Generally, the effect size of a given intervention is bigger when multiple domains and contexts are modified and when social, cultural and environmental aspects are considered [29]. A typical example is a workplace intervention, in which changes in physical environment and targeting the whole workplace community [30], instead of intervening merely at an individual level [31], have been found to be beneficial. The focus on families rather than the workplace community clearly favored leisure time changes, emphasizing the context-specificity of this approach. A recent review identified restructuring of the social or physical environment among the most promising behavior change techniques to reduce sedentary time [32]. In addition, their analysis distinguished different functions across worksite and non-worksite settings, with the idea that worksite sedentary behavior may be more predictable than non-worksite sedentary time [32], which may explain the lack of effectiveness concerning work time in the present study. Even though targeting increased motivation through information provision has been suggested for the pursuit of significant population-level decreases in sedentary behavior [32], the information provision along with the other intervention techniques used in this study affected only leisure time behaviors. However, the present intervention was more effective at increasing women’s work light activity time, whereas a recent meta-analysis reported a more effective reduction in total sedentary time in men [14]. The possible domain- and intervention technique–specific gender effects require further study. Positive group-level long-term changes at work might be achievable by environmental restructuring, which would maximize opportunities for light intensity physical activity alongside work routines, and make the active choice more socially acceptable and even appealing. Even though workplace interventions are considered to be important because the majority of total sitting hours are accumulated at work [33], the present results showed that changes were more effective during leisure time where the sitting time was already lower, and highlight the possibility that leisure sedentary time can be decreased without changes in total or work sedentary time.

In short-term experimental laboratory studies, reallocation of sitting to light intensity activity has been shown to be beneficial for glucose metabolism [34,35], which is in line with the observed decrease in fasting plasma glucose at three months in this study. The proposed acute mechanisms include improved muscle-activity -mediated glucose transport, decreased post-prandial glycemic load and improved plasma triglyceride catabolism among others [34,36,37]. Interestingly, these proposed acute benefits of reducing sedentary time were not reflected during the 12 month follow-up, but beneficial changes in leg lean mass and apoB/apoA-1 ratio were seen. ApoA-1 accounts for the majority of protein in HDL particles and is responsible for the gathering of excess cholesterol into HDL particles from peripheral cells. It also induces anti-inflammatory and antioxidant effects, with apoB inducing atherogenic mechanisms in LDL subclasses. As such, the apoB/apoA-1 -ratio appears to be a better marker for cardiovascular diseases than traditional lipids or lipid ratios [38]. In a study by Duvivier et al. (2013), light intensity physical activity that reduced total sitting time reduced triglycerides and insulin response, but also apoB concentrations [9]. Even though the apoB/apoA-1 -ratio was not reported, their short term result supports our notion that long-term changes in sedentary time may also influence apolipoproteins. Moreover, just two weeks of reduced daily ambulatory activities results in a decline in leg lean mass due to decreased muscle protein synthesis without changes in upper body lean mass, providing some support for the observation of this study [39,40]. Although the changes were small, these novel findings support distinct acute vs. long term benefits of reduced leisure sedentary time even in a group of healthy, normal weight and relatively young individuals.

An unexpected finding was that the cardio-metabolic benefits were evident during the one-year follow-up despite the fact that the intervention did not decrease total sedentary time, and that the decrease in sedentary leisure time plateaued towards the end of the study. Based on epidemiological evidence, the health risks of sedentary leisure time are more pronounced than those of work time, suggesting that a domain-specific decrease in sedentary time can be effective even without decreases in total sedentary time [15,16]. Indeed, the participants were able to decrease leisure sedentary time at the beginning of the study with a sufficient magnitude to produce some cardio-metabolic benefits [34,35]. Even though the decrease in sedentary time plateaued towards the end of the study, beneficial changes in leg lean mass and anthropometrics were evident only at the 12 month time point. Group-specific examination revealed that the beneficial intervention effect on leg lean mass was due to an adverse change within the control group, suggesting that their high sedentary time throughout the year and an increasing trend in sedentary leisure time at the end of the study were hazardous, but even a small decrease in the intervention group’s leisure sedentary time was enough to prevent a decrease in leg lean mass. A similar trend towards a group effect was observable in body mass, BMI and arm and leg fat mass, but the group x time effects were insignificant. These findings should be considered as preliminary since this study was not powered for secondary health outcomes. Moreover, although there were no group x time effects in any diet variables, some within-group changes in energy intake may have contributed to the observed results. Some of the key cardio-metabolic effects of sedentary time may be linked to interactions between sedentary periods and energy intake, and should be carefully assessed in future interventions [41].

Bearing in mind the aforementioned uncertainties, the present data points to a possibility that some health benefits of reducing sedentary time may be attributable to the prevention of adverse health effects of high sedentary time. This idea is supported by experimental findings of rapid deleterious effects of increased sedentary time on insulin sensitivity, maximal oxygen consumption and leg lean mass, which are easy to prevent with habitual levels of physical activity [40]. In addition, compositional data-analyses have shown that an increase in sedentary time is more deleteriously associated with cardio-metabolic health markers than the benefits gained by a decrease in sedentary time of similar magnitude [42]. Future interventions should consider whether simply maintaining the current level of sedentary behavior, which prevents an increase in sedentary time, could be an achievable, feasible and ultimately effective goal at the population level and within the lifespan of an individual, which both show an increasing trend in sedentary time [2,24].

Although widely utilized, a clear drawback of this study was the use of waist-worn accelerometers to assess the primary outcomes. The primary results illustrate changes between non-movement and movement, but provide no information about postures like standing, although standing increases muscle activity and is beneficial for health [10,43]. Another limitation was that the a-priori planned sample size was not reached [18]. However, the significant findings in some of the primary outcomes suggest that the sample was big enough to test the primary hypothesis. The outcomes were assessed objectively with accelerometers at several time points, which is a strength of this study. Moreover, separation of different domains in intervention message and analysis, assessment of energy intake, long follow-up, robust statistical methods and a no-treatment control group enabled assessment of domain-specific behavioral and physiological effectiveness of the intervention, which is relevant for the field. Taken together, the present results provide a conservative estimate of the long-term domain-specific effectiveness of an intervention targeting sedentary-time, which could be improved by assessing posture and including high-risk participant groups.

## Conclusions

This cluster randomized controlled trial targeting reduced sedentary time in parents with a sedentary occupation and young children had a beneficial effect on weekday sedentary leisure time in the intervention group compared to controls without affecting total, weekend or work sedentary time. The behavioral method induced a small decrease in weekday sedentary leisure time throughout the year, and although the magnitude of decrease plateaued towards the end of the study, beneficial effects on apoB/apoA-1 balance and leg muscle mass were observed. In the long term, a change in only sedentary leisure time might induce small health benefits, and can be achieved by behavioral counseling targeting parents with a sedentary occupation and young children. Given the evident challenges in promoting moderate-to-vigorous physical activity, reducing sedentary leisure time may be a feasible alternative to reap health benefits.

## Supporting information

S1 Checklist CONSORT checklist. (DOCX)

S2 Checklist TIDieR checklist. (DOCX)

S1 Table Secondary outcomes at baseline. (DOCX)

S2 Table Intervention effectiveness on energy intake and diet composition. (DOCX)

S3 Table Intervention effectiveness on anthropometrics and blood pressure. (DOCX)

S4 Table Intervention effectiveness on blood-drawn cardio-metabolic biomarkers. (DOCX)

S1 Supporting information Simulation analysis for data reliability. (DOCX)

S1 Protocol InPact study plan for ethics committee. (DOCX)
