# Supplementary material for: Accelerometer-assessed sedentary work, leisure time and cardio-metabolic biomarkers during one year: Effectiveness of a cluster randomized controlled trial in parents with a sedentary occupation and young children
Source: PLoS One. 2017 Aug 24;12(8):e0183299. doi: 10.1371/journal.pone.0183299 (PMC5570316; doi:10.1371/journal.pone.0183299)
Supplement: S1 Table — (DOCX) [file pone.0183299.s003.docx]

**S1 TABLE.** Secondary outcomes at baseline.

|  | Intervention | | Control | |
| --- | --- | --- | --- | --- |
|  | n |  | n |  |
| Energy intake, kcal / d | 59 | 2057 (494) | 55 | 2146 (520) |
| Protein, E% | 59 | 18.2 (3.4) | 55 | 18.1 (3.2) |
| Carbohydrate, E% | 59 | 43.3 (5.6) | 55 | 44.2 (6.6) |
| Fat, E% | 59 | 34.1 (5.6) | 55 | 33.6 (6.4) |
| Saturated fat, E% | 59 | 11.9 (3.1) | 55 | 11.4 (2.8) |
| Monounsaturated fat, E% | 59 | 10.8 (2.3) | 55 | 10.4 (2.9) |
| Polyunsaturated fat, E% | 59 | 4.9 (1.2) | 55 | 4.9 (1.4) |
| Alcohol, g/d | 59 | 5.6 (9.6) | 55 | 4.7 (9.2) |
| Weight, kg | 71 | 72.0 (15.4) | 60 | 71.8 (14.0) |
| BMI, kg/m^2^ | 71 | 24.5 (3.5) | 60 | 24.4 (4.1) |
| Arm fat mass, % | 70 | 2.6 (0.8) | 59 | 2.5 (1.0) |
| Leg fat mass, % | 70 | 10.1 (3.4) | 59 | 9.4 (4.0) |
| Trunk fat mass, % | 70 | 15.0 (4.6) | 59 | 14.1 (4.6) |
| Total fat mass, % | 70 | 28.6 (7.5) | 59 | 26.9 (8.7) |
| Arm lean mass, % | 70 | 7.6 (1.6) | 59 | 8.0 (1.7) |
| Leg lean mass, % | 70 | 22.3 (2.8) | 59 | 23.0 (3.3) |
| Trunk lean mass, % | 70 | 32.1 (3.5) | 59 | 32.9 (4.0) |
| Total lean mass, % | 70 | 67.1 (7.6) | 59 | 69.1 (8.9) |
| Systolic BP, mmHg | 61 | 116.6 (10.7) | 59 | 117.3 (10.4) |
| Diastolic BP, mmHg | 61 | 73.8 (8.0) | 59 | 74.6 (8.4) |
| Total cholesterol, mM | 67 | 4.8 (0.8) | 60 | 4.8 (0.9) |
| HDL cholesterol, mM | 67 | 1.8 (0.5) | 60 | 1.7 (0.4) |
| LDL cholesterol, mM | 67 | 2.6 (0.9) | 59 | 2.7 (0.8) |
| Triglycerides, mM | 67 | 1.0 (0.6) | 59 | 1.0 (1.1) |
| Fasting plasma glucose, mM | 66 | 5.3 (0.5) | 60 | 5.2 (0.5) |
| Fasting serum insulin, pM | 60 | 40.7 (29.1) | 44 | 34.9 (19.7) |
| HOMA-IR | 60 | 1.6 (1.3) | 44 | 1.4 (0.8) |
| HOMA-%B | 60 | 75.6 (44.9) | 44 | 71.8 (41.6) |
| Mean diameter of VLDL, nm | 62 | 35.6 (1.5) | 54 | 35.4 (0.8) |
| Mean diameter of LDL, nm | 62 | 23.7 (0.1) | 54 | 23.7 (0.1) |
| Mean diameter of HDL, nm | 62 | 10.0 (0.3) | 54 | 10.0 (0.2) |
| apoA-1, g/l | 62 | 1.5 (0.2) | 54 | 1.6 (0.2) |
| apoB, g/l | 62 | 0.8 (0.2) | 54 | 0.8 (0.2) |
| Ratio of apoB to apoA-1 | 62 | 0.5 (0.1) | 54 | 0.5 (0.1) |

Data are presented as average (SD). BMI, Body mass index; BP, blood pressure; HDL, high-density lipoprotein; LDL, Low density lipoprotein; HOMA, Homeostasis Model Assessment; IR, insulin resistance; %B, basal insulin secretion.
