# Supplementary material for: Accelerometer-assessed sedentary work, leisure time and cardio-metabolic biomarkers during one year: Effectiveness of a cluster randomized controlled trial in parents with a sedentary occupation and young children
Source: PLoS One. 2017 Aug 24;12(8):e0183299. doi: 10.1371/journal.pone.0183299 (PMC5570316; doi:10.1371/journal.pone.0183299)
Supplement: S2 Table — (DOCX) [file pone.0183299.s004.docx]

**S2 TABLE.** Intervention effectiveness on energy intake and diet composition.

|  |  |  | Mean change  (95% CI) | | | Mean difference in change (95% CI) | Group x Time | |
| --- | --- | --- | --- | --- | --- | --- | --- | --- |
|  | Time | n | Intervention (n = 62) | n | Control (n = 59) | Intervention - Control | P | Adj. P |
| Energy intake  (kcal / day) | 3 m | 54 | **-148 (-283 to -14)*** | 53 | -107 (-245 to 31) | -41 (-234 to 152) | 0.64 | 0.71 |
|  | 6 m | 56 | **-276 (-410 to -142)***** | 51 | -90 (-230 to 50) | -185 (-379 to 8) | 0.31 | 0.05 |
|  | 9 m | 50 | **-180 (-319 to -41)*** | 49 | -126 (-268 to 15) | -53 (-252 to 145) | 0.22 | 0.14 |
|  | 12 m | 54 | -126 (-262 to 9) | 48 | **-176 (-319 to -33)*** | 50 (-147 to 247) | 0.18 | 0.13 |
| Protein (E%) | 3 m | 54 | 0.3 (-1.0 to 1.6) | 53 | -0.6 (-1.9 to 0.7) | 0.9 (-1.0 to 2.7) | 0.48 | 0.26 |
|  | 6 m | 56 | 0.5 (-0.8 to 1.8) | 51 | 1.5 (0.1 to 2.8) | -1.0 (-2.8 to 0.9) | 0.21 | 0.13 |
|  | 9 m | 50 | 0.4 (-0.9 to 1.7) | 49 | 0.3 (-1.1 to 1.7) | 0.1 (-1.8 to 2.0) | 0.34 | 0.62 |
|  | 12 m | 54 | 0.5 (-0.8 to 1.8) | 48 | 0.5 (-0.8 to 1.9) | -0.1 (-2.0 to 1.8) | 0.44 | 0.74 |
| Carbohydrate (E%) | 3 m | 54 | 0.7 (-1.8 to 3.1) | 53 | -0.9 (-3.4 to 1.6) | 1.5 (-2.0 to 5.0) | 0.35 | 0.95 |
|  | 6 m | 56 | 0.4 (-2.1 to 2.8) | 51 | -1.5 (-4 to 1.0) | 1.8 (-1.7 to 5.3) | 0.65 | 0.26 |
|  | 9 m | 50 | 1.1 (-1.4 to 3.6) | 49 | -0.6 (-3.1 to 2.0) | 1.7 (-1.9 to 5.3) | 0.74 | 0.90 |
|  | 12 m | 54 | -1.0 (-3.5 to 1.4) | 48 | -2.2 (-4.8 to 0.3) | 1.2 (-2.3 to 4.8) | 0.85 | 0.96 |
| Fat (E%) | 3 m | 54 | 0.2 (-2.2 to 2.6) | 53 | 1.5 (-0.9 to 4.0) | -1.4 (-4.8 to 2.1) | 0.41 | 0.73 |
|  | 6 m | 56 | 0.4 (-2.0 to 2.7) | 51 | 0.9 (-1.5 to 3.4) | -0.6 (-4.0 to 2.9) | 0.75 | 0.47 |
|  | 9 m | 50 | 0.0 (-2.4 to 2.5) | 49 | 1.2 (-1.3 to 3.7) | -1.2 (-4.7 to 2.3) | 0.86 | 1.00 |
|  | 12 m | 54 | 1.6 (-0.8 to 4.0) | 48 | 2.1 (-0.5 to 4.6) | -0.4 (-3.9 to 3.0) | 0.94 | 1.00 |
| Saturated fat (E%) | 3 m | 54 | -0.2 (-1.4 to 1.1) | 53 | 1.0 (-0.3 to 2.3) | -1.2 (-3.0 to 0.6) | 0.33 | 0.34 |
|  | 6 m | 56 | 0.6 (-0.6 to 1.9) | 51 | 0.2 (-1.1 to 1.5) | 0.4 (-1.3 to 2.2) | 0.66 | 0.56 |
|  | 9 m | 50 | 0.0 (-1.3 to 1.2) | 49 | 0.8 (-0.5 to 2.1) | -0.9 (-2.7 to 0.9) | 0.27 | 0.70 |
|  | 12 m | 54 | 0.0 (-1.2 to 1.2) | 48 | 1.1 (-0.2 to 2.4) | -1.1 (-2.9 to 0.7) | 0.28 | 0.77 |
| Monounsaturated fat (E%) | 3 m | 54 | -0.2 (-1.3 to 1.0) | 53 | **1.4 (0.2 to 2.5)*** | -1.5 (-3.1 to 0.1) | 0.21 | 0.54 |
|  | 6 m | 56 | -0.1 (-1.2 to 1.0) | 51 | 1.1 (0.0 to 2.3) | -1.3 (-2.9 to 0.4) | 0.41 | 0.34 |
|  | 9 m | 50 | 0.3 (-0.9 to 1.5) | 49 | **1.7 (0.5 to 2.9)**** | -1.4 (-3.0 to 0.3) | 0.26 | 0.72 |
|  | 12 m | 54 | **1.6 (0.4 to 2.7)**** | 48 | **1.9 (0.7 to 3.1)**** | -0.4 (-2.0 to 1.3) | 0.26 | 0.66 |
| Polyunsaturated fat (E%) | 3 m | 54 | 0.1 (-0.5 to 0.8) | 53 | 0.3 (-0.3 to 1.0) | -0.2 (-1.1 to 0.7) | 0.20 | 0.28 |
|  | 6 m | 56 | 0.0 (-0.6 to 0.6) | 51 | **0.7 (0.1 to 1.4)*** | -0.7 (-1.6 to 0.2) | 0.34 | 0.26 |
|  | 9 m | 50 | 0.4 (-0.2 to 1.1) | 49 | **0.9 (0.2 to 1.5)*** | -0.4 (-1.3 to 0.5) | 0.50 | 0.37 |
|  | 12 m | 54 | **0.9 (0.3 to 1.5)**** | 48 | **1.0 (0.3 to 1.7)**** | -0.1 (-1.0 to 0.8) | 0.55 | 0.53 |
| Alcohol (g/d) | 3 m | 54 | **-4.1 (-6.3 to -2.0)***** | 53 | **-2.7 (-4.9 to -0.5)*** | -1.5 (-4.5 to 1.6) | 0.48 | 0.43 |
|  | 6 m | 56 | **-3.7 (-5.8 to -1.5)***** | 51 | **-2.3 (-4.5 to 0.0)*** | -1.4 (-4.5 to 1.7) | 0.84 | 0.96 |
|  | 9 m | 50 | **-4.5 (-6.7 to -2.3)***** | 49 | **-2.6 (-4.8 to -0.3)*** | -1.9 (-5.1 to 1.2) | 0.65 | 0.73 |
|  | 12 m | 54 | -2.0 (-4.2 to 0.2) | 48 | -0.3 (-2.6 to 1.9) | -1.7 (-4.8 to 1.5) | 0.76 | 0.78 |

Footnote: P-values indicated as follows: * < 0.05, ** < 0.01 and *** < 0.001. Group x time –interaction P-values are based on likelihood ratios. P = unadjusted P-value, Adj. P = P-value adjusted for age, sex, baseline value, season at baseline (spring/summer/autumn/winter), work time/week, number of children and marital status (single/relationship).
