# Supplementary material for: Accelerometer-assessed sedentary work, leisure time and cardio-metabolic biomarkers during one year: Effectiveness of a cluster randomized controlled trial in parents with a sedentary occupation and young children
Source: PLoS One. 2017 Aug 24;12(8):e0183299. doi: 10.1371/journal.pone.0183299 (PMC5570316; doi:10.1371/journal.pone.0183299)
Supplement: S3 Table — (DOCX) [file pone.0183299.s005.docx]

**S3 TABLE.** Intervention effectiveness on anthropometrics and blood pressure.

|  |  |  | Mean change (95% CI) | | | Mean difference between groups (95% CI) | Group x Time | |
| --- | --- | --- | --- | --- | --- | --- | --- | --- |
|  | Time | n | Intervention (n = 71) | n | Control (n = 60) | Intervention-control | P | Adj. P |
| Weight (kg) | 6 m | 61 | -0.24 (-0.80 to 0.31) | 53 | 0.59 (0.00 to 1.18) | **-0.83 (-1.64 to -0.02)*** | 0.60 | 0.98 |
|  | 12 m | 58 | -0.11 (-0.67 to 0.46) | 54 | **0.84 (0.25 to 1.43)**** | **-0.95 (-1.76 to -0.13)*** | 0.13 | 0.33 |
| BMI (kg/m^2^) | 6 m | 61 | -0.07 (-0.26 to 0.11) | 53 | 0.19 (-0.01 to 0.38) | -0.26 (-0.53 to 0.01) | 0.61 | 0.99 |
|  | 12 m | 58 | 0.00 (-0.19 to 0.19) | 54 | **0.28 (0.08 to 0.47)**** | **-0.28 (-0.55 to -0.01)*** | 0.19 | 0.41 |
| Arm fat mass (%) | 6 m | 62 | -0.05 (-0.1 to 0) | 58 | 0.05 (-0.01 to 0.10) | **-0.09 (-0.17 to -0.02)*** | 0.55 | 0.98 |
|  | 12 m | 52 | 0.03 (-0.02 to 0.08) | 53 | **0.12 (0.07 to 0.18)***** | **-0.09 (-0.17 to -0.02)*** | 0.08 | 0.50 |
| Leg fat mass (%) | 6 m | 62 | **-0.19 (-0.33 to -0.04)*** | 58 | 0.11 (-0.04 to 0.27) | **-0.30 (-0.51 to -0.09)**** | 0.25 | 0.92 |
|  | 12 m | 52 | -0.06 (-0.21 to 0.08) | 53 | 0.10 (-0.05 to 0.26) | -0.17 (-0.38 to 0.05) | 0.07 | 0.08 |
| Trunk fat mass (%) | 6 m | 62 | -0.08 (-0.38 to 0.21) | 58 | 0.06 (-0.26 to 0.38) | -0.14 (-0.58 to 0.29) | 0.23 | 0.23 |
|  | 12 m | 52 | 0.05 (-0.25 to 0.35) | 53 | **0.38 (0.06 to 0.70)*** | -0.33 (-0.76 to 0.11) | 0.68 | 0.60 |
| Total fat mass (%) | 6 m | 62 | -0.33 (-0.77 to 0.12) | 58 | 0.22 (-0.27 to 0.71) | -0.54 (-1.2 to 0.11) | 0.59 | 0.43 |
|  | 12 m | 52 | 0.01 (-0.44 to 0.47) | 53 | **0.61 (0.13 to 1.09)*** | -0.60 (-1.26 to 0.07) | 0.37 | 0.29 |
| Arm lean mass (%) | 6 m | 62 | **0.08 (0.01 to 0.15)*** | 58 | 0.04 (-0.04 to 0.12) | 0.04 (-0.07 to 0.15) | 0.82 | 0.85 |
|  | 12 m | 52 | **0.10 (0.03 to 0.18)**** | 53 | 0.06 (-0.02 to 0.14) | 0.05 (-0.06 to 0.16) | 0.92 | 0.54 |
| Leg lean mass (%) | 6 m | 62 | 0.03 (-0.17 to 0.23) | 58 | -0.10 (-0.32 to 0.11) | 0.13 (-0.16 to 0.42) | 0.43 | 0.13 |
|  | 12 m | 52 | 0.04 (-0.16 to 0.24) | 53 | **-0.44 (-0.65 to -0.22)***** | **0.48 (0.18 to 0.77)**** | **0.021** | 0.11 |
| Trunk lean mass (%) | 6 m | 62 | 0.26 (-0.04 to 0.56) | 58 | -0.22 (-0.55 to 0.11) | **0.48 (0.03 to 0.92)*** | 0.87 | 0.93 |
|  | 12 m | 52 | **0.31 (0.00 to 0.62)*** | 53 | -0.11 (-0.44 to 0.21) | 0.42 (-0.02 to 0.87) | 0.22 | 0.10 |
| Total lean mass (%) | 6 m | 62 | 0.43 (-0.09 to 0.95) | 58 | -0.30 (-0.87 to 0.26) | 0.73 (-0.03 to 1.50) | 0.74 | 0.87 |
|  | 12 m | 52 | 0.50 (-0.03 to 1.02) | 53 | -0.56 (-1.13 to 0.00) | **1.06 (0.29 to 1.83)**** | 0.08 | 0.07 |
| Systolic BP (mmHg) | 6 m | 55 | -0.19 (-1.87 to 1.48) | 53 | 1.08 (-0.67 to 2.83) | -1.27 (-3.69 to 1.15) | 0.38 | 0.72 |
|  | 12 m | 49 | **1.82 (0.13 to 3.51)*** | 47 | **2.53 (0.76 to 4.29)**** | -0.70 (-3.15 to 1.74) | 0.89 | 0.75 |
| Diastolic BP (mmHg) | 6 m | 55 | **-2.29 (-3.45 to -1.13)***** | 53 | **-1.82 (-3.04 to -0.61)**** | -0.47 (-2.15 to 1.21) | 0.17 | 0.19 |
|  | 12 m | 49 | **-1.89 (-3.07 to -0.72)**** | 47 | **-1.38 (-2.61 to -0.15)*** | -0.52 (-2.22 to 1.18) | 0.97 | 0.13 |

Footnote: P-values indicated as follows: * < 0.05, ** < 0.01 and *** < 0.001. Group x time –interaction P-value are based on likelihood ratios. P = unadjusted P-value, Adj. P = P-value adjusted for age, sex, baseline value, season at baseline (spring/summer/autumn/winter), work time/week, number of children, marital status (single/relationship), moderate-to-vigorous activity and energy intake. BMI, Body mass index; BP, blood pressure.
