# Supplementary material for: Accelerometer-assessed sedentary work, leisure time and cardio-metabolic biomarkers during one year: Effectiveness of a cluster randomized controlled trial in parents with a sedentary occupation and young children
Source: PLoS One. 2017 Aug 24;12(8):e0183299. doi: 10.1371/journal.pone.0183299 (PMC5570316; doi:10.1371/journal.pone.0183299)
Supplement: S4 Table — (DOCX) [file pone.0183299.s006.docx]

**S4 TABLE.** Intervention effectiveness on blood-drawn cardio-metabolic biomarkers.

|  |  |  | Mean change (95% CI) | | | Mean difference in change (95% CI) | Group x Time | |
| --- | --- | --- | --- | --- | --- | --- | --- | --- |
|  | Time | n | Intervention (n = 67) | n | Control (n = 60) | Intervention - Control | P | Adj. P |
| Total cholesterol (mM) | 3 m | 63 | -0.07 (-0.20 to 0.06) | 56 | 0.01 (-0.13 to 0.14) | -0.08 (-0.26 to 0.11) | 0.71 | 0.73 |
|  | 6 m | 62 | -0.11 (-0.24 to 0.02) | 55 | -0.04 (-0.17 to 0.1) | -0.07 (-0.26 to 0.11) | 0.89 | 0.69 |
|  | 9 m | 58 | -0.07 (-0.2 to 0.06) | 52 | -0.06 (-0.2 to 0.08) | -0.01 (-0.19 to 0.18) | 0.74 | 0.12 |
|  | 12 m | 59 | -0.09 (-0.22 to 0.04) | 53 | 0.04 (-0.09 to 0.18) | -0.13 (-0.32 to 0.06) | 0.63 | **0.042** |
| HDL cholesterol (mM) | 3 m | 63 | **-0.10 (-0.16 to -0.03)**** | 56 | -0.02 (-0.09 to 0.05) | -0.08 (-0.17 to 0.01) | 0.22 | **0.018** |
|  | 6 m | 62 | **-0.16 (-0.23 to -0.10)***** | 55 | **-0.12 (-0.19 to -0.05)***** | -0.04 (-0.13 to 0.06) | 0.45 | 0.80 |
|  | 9 m | 58 | **-0.18 (-0.24 to -0.11)***** | 52 | **-0.16 (-0.23 to -0.09)***** | -0.02 (-0.11 to 0.08) | 0.37 | **0.005** |
|  | 12 m | 59 | **-0.13 (-0.19 to -0.06)***** | 54 | **-0.12 (-0.19 to -0.06)***** | 0.00 (-0.10 to 0.09) | 0.44 | **0.022** |
| LDL cholesterol (mM) | 3 m | 63 | 0.03 (-0.1 to 0.16) | 56 | 0.05 (-0.09 to 0.19) | -0.02 (-0.21 to 0.18) | 0.43 | 0.54 |
|  | 6 m | 62 | 0.06 (-0.07 to 0.2) | 55 | 0.10 (-0.04 to 0.24) | -0.03 (-0.23 to 0.16) | 0.67 | 0.26 |
|  | 9 m | 58 | 0.11 (-0.02 to 0.25) | 52 | **0.15 (0.01 to 0.29)*** | -0.04 (-0.23 to 0.16) | 0.98 | 0.88 |
|  | 12 m | 59 | 0.03 (-0.1 to 0.17) | 54 | **0.17 (0.03 to 0.31)*** | -0.14 (-0.33 to 0.06) | 0.67 | 0.14 |
| Triglycerides (mM)^a^ | 3 m | 63 | 0.05 (-0.06 to 0.17) | 56 | -0.02 (-0.14 to 0.10) | 0.08 (-0.09 to 0.24) | 0.89 | 0.35 |
|  | 6 m | 62 | -0.01 (-0.13 to 0.10) | 55 | 0.01 (-0.11 to 0.13) | -0.02 (-0.19 to 0.14) | 0.16 | 0.33 |
|  | 9 m | 58 | -0.01 (-0.13 to 0.10) | 52 | 0.00 (-0.13 to 0.12) | -0.01 (-0.18 to 0.16) | 0.98 | 0.96 |
|  | 12 m | 59 | 0.06 (-0.05 to 0.17) | 54 | 0.05 (-0.07 to 0.17) | 0.01 (-0.15 to 0.18) | 0.97 | 0.99 |
| Fasting plasma glucose (mM) | 3 m | 62 | **-0.31 (-0.43 to -0.18)***** | 56 | **-0.13 (-0.26 to 0.00)*** | **-0.18 (-0.35 to 0.00)*** | **0.021** | 0.26 |
|  | 6 m | 61 | **-0.18 (-0.31 to -0.06)**** | 56 | **-0.14 (-0.27 to -0.01)*** | -0.04 (-0.22 to 0.13) | 0.34 | 0.71 |
|  | 9 m | 57 | **-0.17 (-0.3 to -0.05)**** | 52 | 0.05 (-0.09 to 0.18) | **-0.22 (-0.4 to -0.03)*** | 0.06 | 0.50 |
|  | 12 m | 58 | 0.04 (-0.08 to 0.17) | 54 | **0.18 (0.05 to 0.31)**** | -0.14 (-0.32 to 0.04) | 0.09 | 0.68 |
| Fasting serum insulin (pM)^a^ | 3 m | 61 | 0.03 (-5.46 to 5.51) | 43 | 0.58 (-6.04 to 7.2) | -0.55 (-9.15 to 8.05) | 0.27 | 0.09 |
|  | 6 m | 57 | 3.66 (-1.94 to 9.27) | 36 | **10.74 (3.73 to 17.75)*** | -7.08 (-16.06 to 1.9) | 0.57 | 0.28 |
|  | 9 m | 53 | 1.5 (-4.26 to 7.26) | 41 | **7.42 (0.60 to 14.24)*** | -5.92 (-14.84 to 3.01) | 0.28 | 0.78 |
|  | 12 m | 54 | 0.82 (-4.9 to 6.55) | 42 | 2.29 (-4.44 to 9.01) | -1.47 (-10.3 to 7.37) | 0.42 | 0.89 |
| HOMA-IR^a^ | 3 m | 59 | -0.10 (-0.34 to 0.14) | 43 | -0.01 (-0.3 to 0.28) | -0.09 (-0.46 to 0.29) | 0.47 | 0.13 |
|  | 6 m | 55 | 0.05 (-0.19 to 0.3) | 36 | 0.43 (0.12 to 0.73) | -0.37 (-0.77 to 0.02) | 0.62 | 0.43 |
|  | 9 m | 50 | -0.05 (-0.31 to 0.2) | 41 | **0.32 (0.02 to 0.62)*** | **-0.37 (-0.76 to 0.02)*** | 0.16 | 0.53 |
|  | 12 m | 51 | 0.04 (-0.22 to 0.29) | 42 | 0.15 (-0.14 to 0.45) | -0.11 (-0.5 to 0.27) | 0.24 | 0.74 |
| HOMA-%B^a^ | 3 m | 59 | **19.46 (6.98 to 31.95)*** | 43 | 6.85 (-8.05 to 21.74) | 12.62 (-6.81 to 32.05) | **0.042** | **0.026** |
|  | 6 m | 55 | 16.93 (4.16 to 29.7) | 36 | **26.71 (10.95 to 42.47)*** | -9.78 (-30.06 to 10.5) | **0.046** | **0.013** |
|  | 9 m | 50 | 11.32 (-1.9 to 24.53) | 41 | 11.47 (-3.84 to 26.79) | -0.16 (-20.39 to 20.07) | 0.23 | 0.13 |
|  | 12 m | 51 | 3.09 (-10.04 to 16.21) | 42 | -4.84 (-19.97 to 10.28) | 7.93 (-12.1 to 27.96) | 0.23 | 0.19 |
| Mean diameter of VLDL (nm) | 3 m | 58 | 0.02 (-0.28 to 0.32) | 53 | 0.01 (-0.30 to 0.33) | 0.01 (-0.42 to 0.44) | 0.51 | 0.53 |
|  | 6 m | 60 | 0.02 (-0.28 to 0.32) | 53 | 0.06 (-0.25 to 0.38) | -0.04 (-0.47 to 0.39) | 0.35 | 0.06 |
|  | 9 m | 56 | 0.17 (-0.13 to 0.47) | 53 | 0.05 (-0.26 to 0.37) | 0.11 (-0.32 to 0.55) | 0.88 | 0.33 |
|  | 12 m | 59 | -0.10 (-0.39 to 0.20) | 54 | 0.04 (-0.27 to 0.35) | -0.14 (-0.57 to 0.29) | 0.85 | 0.39 |
| Mean diameter of LDL (nm) | 3 m | 58 | -0.02 (-0.06 to 0.02) | 53 | 0.03 (-0.01 to 0.07) | -0.05 (-0.1 to 0.01) | 0.12 | 0.14 |
|  | 6 m | 60 | -0.02 (-0.06 to 0.02) | 53 | 0.01 (-0.03 to 0.05) | -0.03 (-0.08 to 0.02) | 0.57 | 0.69 |
|  | 9 m | 56 | -0.04 (-0.07 to 0) | 53 | 0.01 (-0.03 to 0.05) | -0.05 (-0.1 to 0.01) | 0.22 | 0.25 |
|  | 12 m | 59 | **-0.05 (-0.09 to -0.02)**** | 54 | -0.03 (-0.07 to 0.01) | -0.03 (-0.08 to 0.03) | 0.38 | 0.35 |
| Mean diameter of HDL (nm) | 3 m | 58 | 0.00 (-0.04 to 0.04) | 53 | 0.01 (-0.03 to 0.05) | -0.01 (-0.06 to 0.04) | 0.70 | 0.51 |
|  | 6 m | 60 | -0.01 (-0.05 to 0.02) | 53 | -0.01 (-0.04 to 0.03) | -0.01 (-0.06 to 0.04) | 0.66 | 0.14 |
|  | 9 m | 56 | -0.02 (-0.06 to 0.02) | 53 | 0.00 (-0.03 to 0.04) | -0.02 (-0.08 to 0.03) | 0.84 | 0.50 |
|  | 12 m | 59 | -0.01 (-0.05 to 0.03) | 54 | -0.02 (-0.06 to 0.02) | 0.01 (-0.04 to 0.07) | 0.67 | 0.41 |
| apoA-1 (g/l) | 3 m | 58 | 0.01 (-0.03 to 0.05) | 53 | 0.00 (-0.04 to 0.03) | 0.01 (-0.04 to 0.07) | 0.27 | 0.37 |
|  | 6 m | 60 | 0.01 (-0.02 to 0.05) | 53 | -0.01 (-0.05 to 0.02) | 0.03 (-0.03 to 0.08) | 0.77 | 0.41 |
|  | 9 m | 56 | 0.00 (-0.04 to 0.04) | 53 | 0.00 (-0.04 to 0.03) | 0.01 (-0.05 to 0.06) | 0.77 | 0.87 |
|  | 12 m | 59 | **0.05 (0.01 to 0.09)**** | 54 | 0.00 (-0.04 to 0.03) | **0.05 (0.00 to 0.11)**** | 0.25 | 0.60 |
| apoB (g/l) | 3 m | 58 | -0.02 (-0.04 to 0.01) | 53 | 0.01 (-0.02 to 0.04) | -0.02 (-0.07 to 0.02) | 0.60 | 0.98 |
|  | 6 m | 60 | 0.00 (-0.03 to 0.03) | 53 | 0.01 (-0.02 to 0.04) | -0.01 (-0.05 to 0.03) | 0.83 | 0.51 |
|  | 9 m | 56 | -0.01 (-0.03 to 0.02) | 53 | 0.01 (-0.02 to 0.04) | -0.01 (-0.05 to 0.03) | 0.67 | 0.77 |
|  | 12 m | 59 | 0.00 (-0.03 to 0.03) | 54 | 0.03 (0 to 0.06) | -0.03 (-0.07 to 0.01) | 0.60 | 0.27 |
| Ratio of apoB to apoA-1 | 3 m | 58 | -0.02 (-0.03 to 0.00) | 53 | 0.01 (-0.01 to 0.03) | -0.02 (-0.05 to 0.00) | 0.12 | 0.41 |
|  | 6 m | 60 | -0.01 (-0.02 to 0.01) | 53 | 0.01 (-0.01 to 0.03) | -0.02 (-0.04 to 0.01) | 0.63 | 0.27 |
|  | 9 m | 56 | -0.01 (-0.02 to 0.01) | 53 | 0.01 (-0.01 to 0.03) | -0.01 (-0.04 to 0.01) | 0.35 | 0.50 |
|  | 12 m | 59 | **-0.02 (-0.04 to 0.00)*** | 54 | **0.02 (0.00 to 0.04)*** | **-0.04 (-0.07 to -0.01)**** | **0.039** | **0.028** |

Footnote: P-values indicated as follows: * < 0.05, ** < 0.01 and *** < 0.001. Group x time –interaction P-value are based on likelihood ratios. P = unadjusted P-value, Adj. P = P-value adjusted for age, sex, baseline value, season at baseline (spring/summer/autumn/winter), work time/week, number of children, marital status (single/relationship), moderate-to-vigorous activity and energy intake. ^a^P-value testing performed for log-transformed data but non-transformed estimated marginal means are presented. HDL, high-density lipoprotein; LDL, Low density lipoprotein; HOMA, Homeostasis Model Assessment; IR, insulin resistance; %B, basal insulin secretion.
