# Supplementary material for: Accelerometer-assessed sedentary work, leisure time and cardio-metabolic biomarkers during one year: Effectiveness of a cluster randomized controlled trial in parents with a sedentary occupation and young children
Source: PLoS One. 2017 Aug 24;12(8):e0183299. doi: 10.1371/journal.pone.0183299 (PMC5570316; doi:10.1371/journal.pone.0183299)
Supplement: S1 Supporting information — (DOCX) [file pone.0183299.s007.docx]

S1 Supporting information

**Simulation analysis for data reliability**

*In: Accelerometer-assessed sedentary work, leisure time and cardio-metabolic biomarkers during one year: Effectiveness of a cluster randomized controlled trial in parents with a sedentary occupation and young children*

Requirements for a valid accelerometer data were decided based on a simulation analysis to ensure optimal reliability for the sedentary time (% of measurement time) in the present dataset (Table 1). At baseline, measurements including full seven days of data were included, and every possible combination of number of weekdays and weekend days was formulated by randomly removing 1-6 different days from the analysis. The correlation coefficient of % sedentary time between the incomplete data and the original data was calculated to yield reliability of the incomplete measurement. This process was done for each combination and the process was repeated 1000 times. Thus for every combination, 1000 correlation coefficients were calculated and the average of these is presented in Table 1. To estimate reliability, the square of this averaged correlation coefficient was used. A moderate reliability of >0.6 was accepted for a combination to be included into the analysis meaning that a minimum of two days were accepted from which at least one needed to be a weekday (Table 1). In addition, a wear time criteria per day was set to 10 hours based on previous research [1,2].

**TABLE 1.** Reliability (r^2^) of sedentary time (%) measured at the baseline of InPact based on a simulation analysis, where the correlation coefficient (r) between simulated incomplete days and complete seven days measurements were calculated. A minimum of two days measurement including at least one weekday, as emphasized with bold letters, ensured accepted reliability of >0,6.

| Weekend days (n) | Weekdays (n) | | | | | |
| --- | --- | --- | --- | --- | --- | --- |
|  | 5 | 4 | 3 | 2 | 1 | 0 |
| 2 | **0.98** | **0.95** | **0.91** | **0.85** | **0.74** | 0.54 |
| 1 | **0.95** | **0.93** | **0.88** | **0.82** | **0.68** | 0.39 |
| 0 | **0.89** | **0.86** | **0.80** | **0.72** | 0.55 | 0.00 |

References

1. Troiano RP, Berrigan D, Dodd KW, Mâsse LC, Tilert T, Mcdowell M. Physical activity in the United States measured by accelerometer. Med Sci Sports Exerc. 2008;40: 181–188. doi:10.1249/mss.0b013e31815a51b3

2. Matthews CE, George SM, Moore SC, Bowles H, Blair A, Park Y, et al. Amount of time spent in sedentary behaviors and cause-specific mortality in US adults. Am J Clin Nutr. 2012;95: 437–445. doi:10.3945/ajcn.111.019620.Am
